# The Bismuth Skiagram in Investigating Gastro Intestinal Disorders

**Published:** 1907-11-23

**Authors:** 


### Points in Diagnosis.

### THE BISMUTH SKIAGRAM IN INVESTIGATING GASTRO-INTESTINAL DISORDERS.

We should be sorry to see the $x$-rays resorted to before other measures for arriving at a diagnosis had been tried first. We feel strongly that hospital students nowadays learn to rely too much upon $x$-rays in cases of bony fractures, to the detriment of their future skill in examining cases where no $x$-rays are at hand to help them; and if $x$-rays were used in a similar routine way in the examination of the alimentary canal in hospital cases, it might ultimately be to the detriment rather than the benefit of the patients whom each student will have under his care in his practice subsequently. Nevertheless, our methods of determining certain points about the stomach and intestines are not so exact that we can afford to do without any help that the $x$-rays can give us; and there is much evidence to show that, suitably employed in particular cases, the assistance we can get from them is very great.

### The Essentials of the Method.

The principle upon which the method is based is that of giving the patient a mixture in which a bismuth salt is suspended in a mucilage; the bismuth is precipitated within the alimentary canal, and the organ in which it is so precipitated gives a dark shadow both on the $x$-ray screen and in an $x$-ray photograph. The best salt to use is the carbonate, from which no ill effects accrue; the subnitrate is liable to cause formation of nitrites, with somewhat unpleasant results in a few cases.

It is essential that the stomach or intestines, as the case may be, should be as free from food or fæces as possible at the time the $x$-ray bismuth picture is being taken. Fæces in the large intestine throw all sorts of shadows, and the picture may be still more confused if very clear areas due to gas in the bowel alternate with darker areas due to fæces. This is well known in cases where a kidney is being examined for a calculus; if the bowel has not been emptied before the skiagram is taken, an opacity in the photograph may suggest a renal calculus, though it may really be a scybalous mass in front of the kidney in the ascending or the descending colon. Very soon after the bismuth mixture has been swallowed the precipitated bismuth forms a uniform coating to the inside of the stomach, and the $x$-rays show the exact outline of this organ; after a longer or shorter interval the bismuth begins to pass through the pylorus into the duodenum, and a measure of the mobility of the stomach can thereby be obtained. In cases of pyloric obstruction, the great delay in the passage of the bismuth onwards is a very valuable additional means of diagnosis. After a certain number of hours the bismuth should all be in the colon, and it may here be investigated with the $x$-rays again, if necessary.

### The Method Described.

The $x$-ray examination by means of the bismuth shadow is applicable to every portion of the alimentary canal below the pharynx. The stomach and intestines can be studied more completely and more accurately by this method than has heretofore been possible. Marked prolapse may be detected when other physical examinations fail to show it.

The technique varies in the hands of different observers. As a rule bismuth carbonate is the salt used, suspended in mucilage, and added to a bowl of bread and milk; and from 1 to 4 oz. of this salt are given in from 6 to 30 oz. of total bulk of bread and milk. The patient finds no difficulty in taking it, though naturally he would not be asked to do so unless his illness made it well worth while pursuing this particular method of investigating it. In some cases the bismuth is given through a small-bored stomach tube, and then, although ill-effects are not to be anticipated from the quantity of bismuth used, the salt can be removed from the stomach by means of the same tube after the $x$-ray picture has been obtained. The patient should hold his breath for eight to fifteen seconds if a photograph is taken, to minimise any blurring that might arise from movements of the diaphragm.

If it is desired to investigate the mobility of the stomach, or the condition of the colon, the bismuth must naturally not be siphoned off again. It is then usual to take three skiagrams at intervals; the first is taken within half an hour after the bismuth is administered; the second six hours later, no solid food being allowed in the meantime; the third, twelve to fourteen hours after the first, this being the best time for pictures of the colon. The first skiagram shows the size, shape, and position of the stomach; the second affords a measure of the mobility and emptying power of the stomach, and the third shows the position, size, and shape of the colon.

### Its Limitations.

It will be hardly necessary to indicate the kinds of conditions in which such $x$-ray pictures are likely to prove of value. Every practitioner has cases under his care in whom he would be glad to know the things which the bismuth skiagram can tell him. The surgeon finds the pictures of the greatest assistance previous to operation. The permanency of the record and the evidence afforded of the effects of treatment add value to the method. Skiagrams have proved serviceable in the diagnosis of stricture, dilatation and sacculation of the œsophagus; of dilatation and prolapse of the stomach; of hour-glass stomach; of pyloric obstruction by healed ulcer or by growth; of stricture, dilatation, and tumours of the intestine; of prolapse of the colon and sigmoid flexure.

Like all clinical methods, this one also has its limitations, of course; and it would always be unwise to rely upon it solely in any particular case. As an additional means of diagnosis, however, it is worthy of being well known, for it is undoubtedly of considerable service.

